# Epistaxis in Visceral Leishmaniasis with Hematological Correlation

**DOI:** 10.1155/2012/809056

**Published:** 2012-01-04

**Authors:** B. Sigdel, S. Bhandary, S. Rijal

**Affiliations:** ^1^Department of Otorhinolaryngology and Head and Neck Surgery, Gandaki Medical College, Pokhara, Nepal; ^2^Department of Otorhinolaryngology and Head and Neck Surgery, B. P. Koirala Institute of Health Sciences, Dharan, Nepal; ^3^Department of Internal Medicine, B. P. Koirala Institute of Health Sciences, Dharan, Nepal

## Abstract

*Objective*. To study the prevalence of epistaxis in visceral leismaniasis and its correlation with hematological profile. *Methods*. Out of 80 diagnosed cases of visceral leishmaniasis, 19 patients with epistaxis were included in the study. Diagnosis was made by Rk-39 from peripheral smear and LD bodies from bone marrow. Before starting anti-kala-azar treatment, nasal examination findings and hematological profile were noted. *Study Design*. Prospective cross-sectional hospital-based study. *Results*. Epistaxis was found in the age group of 7–66 years. Epistaxis was observed in 19 (23.8%) cases. One patient died because of epistaxis and neck hematoma. *Conclusion*. Epistaxis is a common ENT finding in endemic area of visceral leishmaniasis like our case.

## 1. Introduction

Leishmaniasis refers to the spectrum of diseases caused by *Leishmania* species, which are Protozoa of order Kinetoplastida. Clinically leishmaniasis is divided into visceral (kala-azar), cutaneous, mucosal, and mucocutaneous syndromes and PKDL. Kala-azar is most commonly caused by *L. donovani*, *L. infantum,* and *L. chagasi* [1–3].

Visceral leishmaniasis has been reported from >60 countries [3]. An estimated 500 000 persons are affected by visceral leishmaniasis every year worldwide. The vast majority of these cases (90%) occur in poor rural area of India, Bangladesh, Sudan, Brazil, and Nepal [4, 5]. *L. donovani* is responsible for visceral leishmaniasis in eastern India, Bangladesh, Nepal, and vast areas of East Africa [1, 3].

Visceral leishmaniasis (VL) is a major public health problem in Nepal. The disease is endemic in twelve terai districts of eastern and southern Nepal. It is estimated that more than 6 million people in Nepal are at risk of leishmania infection [1].

Different otorhinolaryngological manifestations are seen in leishmaniasis. One of the common findings is epistaxis. Prevalence of epistaxis in visceral leishmaniasis up to 51% was found in Sudan and the Mediterranean littoral area [6]. The exact prevalence and cause of epistaxis was not clear in our setup. This study was done to find out the prevalence and hematological risk factor of epistaxis among VL patients.

## 2. Materials and Methods

This study was conducted at Department of Otolaryngology and Head and Neck Surgery and Department of Internal Medicine in B. P. Koirala Institute of Health Sciences Dharan, Nepal over a period of one year from January 2007 to January 2008. It was a hospital-based prospective cross-sectional study. The total of 80 cases of VL was followed in the study period. VL was diagnosed based on clinical profile and the presence of Rk-39 from peripheral smear and LD bodies from bone marrow or tissue biopsy. Before starting anti-kala-azar treatment, nasal examination finding and hematological profile were noted. The other appropriate diagnostic test was done as necessary. Finally, data analysis was done by using SPSS 12.0 version.

**Inclusion criteria were the following.**

- All the diagnosed cases of visceral leishmanias that presented with epistaxis.
- Either of sex.

**Exclusion criteria were the following.**

- Patient already on anti-VL treatment.
- Any other established condition/comorbidities giving rise to similar manifestation.
- Patient not willing to participate in the study.

## 3. Results

This study includes a total of 80 diagnosed cases of visceral leishmaniasis. Nasal bleeding was found in 19 (23.8%) cases with age ranging from 7 to 66 years and median age 31.85 ± 12.15 years. 13 (68.5%) cases were males and 6 (31.5%) were females. Male-to-female ratio was 2.17 : 1 (Table 1). Unilateral bleeding was seen in 11 (57.9%) cases, whereas bilateral bleeding was seen in 8 (42.1%) cases (Table 2). Fourteen (74%) cases were anterior type and 5 (26%) were posterior (Table 3). Sixteen (84.2%) of epistaxis patients had Hb <10 gm/dL with mean 8.1 gm/dL, 17 (89.5%) had total leucocyte count <4000 with mean 2940/mm^3^, and 19 (100%) had platelet count <150 0000 with mean 81526/mm^3^ at presentation (Table 4). Mean PT was 16.5 and INR 1.35. Four (21.1%) had INR >1.6 at presentation. On univariate analysis epistaxis was significantly correlated with thrombocytopenia (*P* = 0.021) (Table 5).

## 4. Discussion

Leishmaniasis is caused by infection with parasites of the genus *Leishmania*. Leishmaniasis is not a single disease but a “variety of syndromes” that are complex and cosmopolitan [6]. Visceral leishmaniasis is caused by *L. donovani* which is endemic in eastern terai of Nepal [7].

Different types of ENT manifestation were found in visceral leishmaniasis. One of the common manifestations is epistaxis. Prevalence of epistaxis in visceral leishmaniasis was around 51% in Sudan and the Mediterranean littoral area [6] and 47–88% in the study of Zijlstra and EL-Hassan [8]. In our study it was only 23.8%, it may be due to the different zoographical locations and hospital-based study.

Bilateral bleeding occured in 11 (57.9%) of cases and unilateral in 8 (42.1%). Most of the bleeding was of the anterior type (14 (73.7%) cases). There was no specific pattern of bleeding as most of cases had diffuse mucosal bleed.

Anterior epistaxis refers to bleeding point seen on anterior rhinoscopy. Posterior bleeding refers to active bleeding refractory to adequate anterior packing or when no bleeding point is identified on anterior rhinoscopy [9].

The pathogenesis of epistaxis occurring in early phase of disease is not understood, but that occurring late in the disease is probably due to a combination of deficient clotting factor and thrombocytopenia [6, 10].

In our study out of 19 epistaxis patients, 84.2% had Hb <10 gm/dL, 89.5% had TLC <4000/mm^3^, and 100% had platelet count <150 000/mm^3^.

The cause of pancytopenia is multifactorial. Sequestration and destruction of erythrocyte [11] and haemolysis lead to shortened half-life, iron deficiency, and folate deficiency reported [11–13]. Immune lysis and ineffective erythropoiesis may contribute to the anaemia. Neutrophil and platelets are sequestrated and destroyed prematurely [6]. But it is not known whether the observed neutropenia and thrombocytopenia is due to increased margination, splenic sequestration, or an autoimmune process or combination of those factors [3].

Mean prothrombin time was 16.60 ± 2.8 (range 12–28). INR was 1.28 ± 0.2 (range 1.0–2.0). Renal function test (serum urea and creatine) was within normal limits.

Among the patients with epistaxis, mean PT was 16.5 and INR 1.35. Four (21.1%) had INR >1.6 at presentation. Marginally prolonged prothrombin time has been reported by EL-Hassan et al. [11].

 Epistaxis was correlated with different hematological parameters like haemoglobin, TLC, platelet count, PT, and INR. Epistaxis was significantly correlated with thrombocytopenia (*P* = 0.02).

In our study, all patients were managed initially with nasal decongestant, though 17 (89%) required chemical cautery, nasal packing with abgel. In 7 patients anterior nasal packing and in 5 patients posterior nasal packing using Foley catheter were done for the control of nasal bleeding followed by blood transfusion.

One 23/M had neck hematoma with nasal bleeding. Incision and drainage were done for neck hematoma. Posterior nasal packing was done for nasal bleeding. In spite of blood transfusion and packing, the patient died due to excessive uncontrollable bleeding.

Bleeding from the anterior nares is one of the least understood symptoms of VL. The bleeding may be severe and life threatening. The presence of parasites in the nasal mucosa may play a role. The bleeding usually responds well to symptomatic therapy (nasal tamponade using gauze with 1% lignocaine) but it often recurs [8].

## 5. Conclusion

Epistaxis is a common ENT finding in endemic area of visceral leishmaniasis. Pancytopenia is a common laboratory finding. Epistaxis may be due to thrombocytopenia. Further studies with larger series of cases and followup are needed to be taken into consideration.

## Figures and Tables

**Table 1:** Gender distribution.

Gender	No. of cases	Percent (%)
Male	13	68.5
Female	6	31.5

Total	19	100

**Table 2:** Site of epistaxis.

Laterality	No. of cases	Percent (%)
Unilateral nostril	11	57.9
Bilateral nostril	8	42.1

Total	19	100

**Table 3:** Classification of epistaxis.

Classification	No.	Percent (%)
Anterior	14	74
Posterior	5	26

Total	19	100

**Table 4:** Hematological profile of epistaxis patients.

Haemoglobin			Total leucocyte count			Platelet count		
Hb (gm/dL)	No. of cases	%	TLC (/mm^3^)	No. of cases	%	Platelet count (/mm^3^)	No. of cases	%
>10	3	15.8	>4000	2	10.5	>150 000	0	0
<10	16	84.2	<4000	17	89.5	<150 000	19	100

Total	19	100	Total	19	100	Total	19	100

Mean Hb = 8.1 (range 5.6–10.56) gm/dL			Mean TLC = 2940			Mean platelet count = 81526		

**Table 5:** Univariate analysis: risk factor for epistaxis.

Variable	Odds ratio	95% confidence interval		*P* value
		Lower	Upper	
Hb <10 gm/dL	1.9	0.48	7.39	0.27
TLC <4000/mm^3^	1.44	0.42	4.99	0.39
Platelet count <150 000	1.39	1.20	1.62	0.02*
PT >14 sec	1.11	0.31	3.91	0.56
INR >1.6	1.82	0.53	6.20	0.33

*P value < 0.05 significant.
